# Hyperpigmentation of the Tongue

**Published:** 2014-01-01

**Authors:** Lindsay Lee Stringer, Laura Zitella

**Affiliations:** From Stanford Hospital and Clinics, Palo Alto, California

## History

Mrs. T. is a 28-year-old woman of East Asian descent. She had been in good health, with no significant medical or surgical history, until she developed low-grade fever, fatigue, and weight loss followed by headaches, orthopnea, and cough. She presented to the emergency department with right neck and facial swelling.

A CT scan of her thorax showed a large soft-tissue mass in the inferior mediastinum. PET/CT showed an anterior mediastinal mass and enlarged hypermetabolic lymph nodes in the subcarinal, paratracheal, para-aortic, and hilar areas. Fine-needle aspiration of the mass confirmed stage IIB primary mediastinal B-cell lymphoma. Bone marrow biopsy showed no evidence of disease, and her lactate dehydrogenase (LDH) level was elevated at 413 IU/L.

Mrs. T.’s treatment plan consisted of 6 cycles of the dose-adjusted R-EPOCH (DA-R-EPOCH) regimen (Wilson et al., 2002; 2008). This every-3-week regimen includes rituximab (Rituxan), etoposide, prednisone, vincristine, cyclophosphamide, and doxorubicin, with etoposide, vincristine, and doxorubicin infused continuously over 96 hours. The doses of etoposide, cyclophosphamide, and doxorubicin are adjusted based on the absolute neutrophil count and platelet nadirs to individualize and maximize dosing. Mrs. T. tolerated the first 2 cycles well. The etoposide, cyclophosphamide, and doxorubicin doses were increased by 20% with each cycle based on her nadir lab values. She was admitted to the hospital for cycle 3 of the chemotherapy regimen.

## Chief Complaint

Mrs. T. reported new-onset bilateral paresthesia in her fingers, which was intermittent and uncomfortable but not painful. Her fine motor skills were intact. After her last treatment cycle, she had fatigue and nausea that resolved within a week. She had mild hypogeusia but an unchanged appetite. She denied oral or throat pain, oral ulcerations, dysphagia, and odynophagia. She had no shortness of breath or chest pain, and system review was otherwise normal.

## Physical Examination

Mrs. T. had multiple dark brown hyperpigmented macules on the dorsal surface of her tongue (see Figure). There was no mucositis or posterior oropharyngeal erythema. The buccal mucosa and gingiva were without lesions, with normal papillae structure on the tongue. She had moderate brown skin (Fitzpatrick type IV) that had slightly darkened since she began chemotherapy. There were multiple café au lait–colored macules of 8 to 13 mm on her palms and the plantar surfaces of her feet. Her nail beds were darkened at the bases. She had no palpable lymphadenopathy in her cervical and supraclavicular regions. Her physical exam was otherwise unremarkable, and her weight was unchanged.

**Figure 1:**
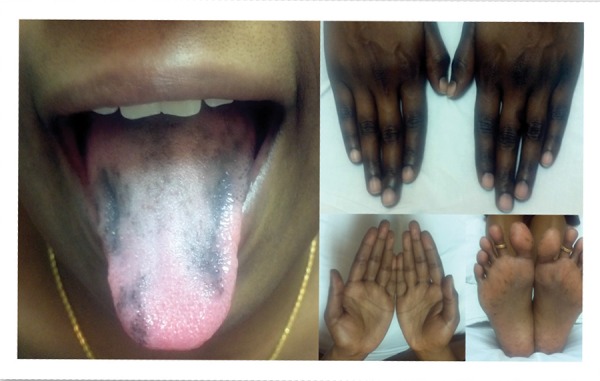

Mrs. T. was afebrile and normotensive, with all vital signs stable. Her complete blood cell count showed mild anemia, with a hemoglobin of 11 g/dL and a hematocrit of 33%. Her white blood cell count was 6.7 × 103/ìL, and her platelet count was 250 billion/L. Her comprehensive metabolic panel was within normal limits, and her LDH level was 288 IU/L.

**Figure 2:**
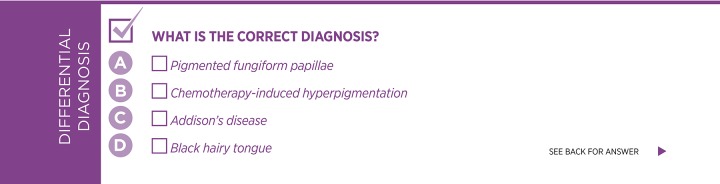
What Is The Correct Diagnosis?

## Correct Answer: B

**Chemotherapy-induced hyperpigmentation.** Hyperpigmentation of the tongue has been reported with both doxorubicin and cyclophosphamide, two of the agents in DA-R-EPOCH. Darkened patches on the tongue may be seen after 1 or 2 chemotherapy treatments. These changes may be localized or widespread and may alter the pigmentation of mucous membranes, skin, or nails. The pathogenesis for this change is not known. The hyperpigmentation begins to fade after chemotherapy is completed and usually disappears within weeks to months (Abbasi & Wang, 2008; Blaya & Saba, 2011; Krutchik & Buzdar, 1979).

## Explanation of Incorrect Answers

**Pigmented fungiform papillae **are a normal variant of the tongue that has no associated pathologic significance. The pigmentation usually presents in late childhood and does not progress or change over time (Mukamal et al., 2011). This finding is more common in individuals with dark-pigmented skin but may appear in any race. The most common presentation is a pattern of diffuse patches on the dorsal lingual surface. The patches may also be seen on the anterior-lateral surface, at the tip of the tongue. The color variation of these patches may range from brown to dark black (Holzwanger, Rudolph, & Heaton, 1974), but there are no associated skin, nail, or other cutaneous changes (Romiti & Molina De Medeiros, 2010). In this case, Mrs. T.’s dark complexion and the pattern of hyperpigmentation on her tongue could represent pigmented fungiform papillae. However, given that this is a new finding in a 28-year-old woman who also has associated skin and nail changes, this diagnosis is unlikely.

Addison’s disease. Hyperpigmentation, which occurs in nearly all cases of Addison’s disease is most prominent in areas of increased friction, such as the palmar creases, the interphalangeal joints, and the oral mucosa. With adrenal insufficiency, a compensatory activation of the hypothalamic-pituitary axis results in high plasma adrenocorticotropic hormone (ACTH) and melanocyte-stimulating hormone concentrations. This hormonal change is responsible for the hyperpigmentation. Other common symptoms of Addison’s disease include fatigue, anorexia, nausea and vomiting, and abdominal pain. In addition, hypotension, hypoglycemia, hyponatremia, and hyperkalemia are often observed (Barnard et al., 2004; Fauci et al., 2009). Mrs. T. reported fatigue and nausea that resolved spontaneously, which would be most consistent with delayed chemotherapy-induced nausea rather than Addison’s disease. In addition, because her lab values and blood pressure were normal, Addison’s disease is unlikely.

Black hairy tongue. Lingua villosa nigra, also known as black hairy tongue, is characterized by a black discoloration and hairy texture on the dorsal surface of the tongue. This benign change occurs when defective debridement of the tongue results in an accumulation of bacteria, fungus, and food on the tongue surface. The underlying mechanism for change is unknown, but possible associated factors include broad-spectrum antibiotics, psychotropic medications (benzodiazepines and antidepressants), oxidizing mouthwashes (sodium peroxide and hydrogen peroxide), tobacco, and alcohol. Nausea, a gagging sensation, and halitosis are potential accompanying symptomatic side effects. Although its physical appearance is alarming, black hairy tongue has no long-term adverse effects. Treatment includes increased hydration; proper oral hygiene; cessation of smoking; and, occasionally, surgical excision of the filiform papilla for symptomatic relief and cosmetic purposes (Korber & Dissemond, 2006; Thompson & Kessler, 2010). This is an unlikely diagnosis for Mrs. T. because she presented with diffuse patchy hyperpigmentation rather than a black coating over her tongue.

## Conclusion

Skin hyperpigmentation can be a distressing side effect of chemotherapy. The role of the advanced practitioner (AP) is to educate patients about this potential side effect prior to the initiation of treatment. The AP can also reassure patients that these changes will likely disappear within weeks to months after completion of treatment.
